# Prevalence and Significance of Approximate Symmetry in *Z’* > 1 Orgainc Structures

**DOI:** 10.1063/4.0001038

**Published:** 2025-10-27

**Authors:** Carolyn P Brock

**Affiliations:** University of Kentucky, Lexington, KY, USA

## Abstract

Approximate symmetry that is periodic in at least one dimension has been found to be pervasive in organic structures with *Z’* > 1 in space groups *P*1,^1^ *C*2,^2^ *Pc*,^3^ and now *Cc*, and in all space groups when *Z’* > 4.^4^ The four space groups considered are the four most common triclinic and monoclinic groups for which the number of archived structures is less than 10^4^. Structures in those groups were included in the surveys if *R* ≤ 0.050.

The approximate symmetry is usually obvious if the right view is found. It is usually easy to distinguish between excellent and too-good approximate symmetry.

Different space groups are associated with different types of approximate symmetry, but approximate inversion centers are common in all four groups.

While some of these structures were determined below a temperature-driven phase transition from a higher-symmetry phase, in most cases the required changes seem too large (*e.g.*, more than 2° in a cell angle) for such a transition. Another possibility is nucleation in a higher-symmetry group followed by distortion during crystal growth.

In the case of approximate 2D symmetry adjacent layers are very often offset by *ca*. **a**_layer_/4. Illustrations show that such an offset allows the layers to fit together better than they would if the approximate symmetry were 3D.

## Figures and Tables

**Table:** 

	SG #1 *P*1	SG #5 *C*2, *A*2, *1*2	SG #7 *Pc, Pn*, *Pa*	SG #9 *Cc, An*, *la* (so far)
All structures examined after rejection of incomplete and duplicate structures	1456	570	377	531
Space group assignment probably incorrect	8%	2%	2%	5%
All reliable structures	1337	557	347	504
Racemic or achiral	18%	15%	(all)	(all)
Have approximate 3D (*i.e.,* space-group) symmetry (includes approximate translations)	48%	43%	44%	23%
Have approximate 2D (*i.e.,* layer-group) symmetry	27%	8%	21%	40%
Have approximate 1D (*i.e.,* rod-group) symmetry	1%	6%	8%	6%
Have approximate inversion centers	30%	17%	33%	36%
Some 2D regions have approximate symmetry	10%	4%	3%	6%
Local approximate symmetry relates layers	0%	11%	7%	0%
No approximate symmetry found	14%	27%	17%	24%
